# Risk factors of treatment failure and 30-day mortality in patients with bacteremia due to MRSA with reduced vancomycin susceptibility

**DOI:** 10.1038/s41598-018-26277-9

**Published:** 2018-05-18

**Authors:** Chien-Chang Yang, Cheng-Len Sy, Yhu-Chering Huang, Shian-Sen Shie, Jwu-Ching Shu, Pang-Hsin Hsieh, Ching-Hsi Hsiao, Chih-Jung Chen

**Affiliations:** 1 College of Medicine, Chang Gung University, 333 Taoyuan, Taiwan; 2 Division of Infectious Diseases, Department of Internal Medicine, Chang Gung Memorial Hospital, 333 Taoyuan, Taiwan; 3 Division of Infectious Diseases, Department of Internal Medicine, Kaohsiung Veterans General Hospital, Kaohsiung, Taiwan; 4 Division of Pediatric Infectious Diseases, Department of Pediatrics, Chang Gung Memorial Hospital, 333 Taoyuan, Taiwan; 5 Department of Medical Biotechnology and Laboratory Science, College of Medicine, Chang Gung University, 333 Taoyuan, Taiwan; 6 Department of Orthopaedic Surgery, Chang Gung Memorial Hospital, 333 Taoyuan, Taiwan; 7 Department of Ophthalmology, Chang Gung Memorial Hospital, 333 Taoyuan, Taiwan

## Abstract

Bacteremia caused by MRSA with reduced vancomycin susceptibility (MRSA-RVS) frequently resulted in treatment failure and mortality. The relation of bacterial factors and unfavorable outcomes remains controversial. We retrospectively reviewed clinical data of patients with bacteremia caused by MRSA with vancomycin MIC = 2 mg/L from 2009 to 2012. The significance of bacterial genotypes, *agr* function and heterogeneous vancomycin-intermediate *S*. *aureus* (hIVSA) phenotype in predicting outcomes were determined after clinical covariates adjustment with multivariate analysis. A total of 147 patients with mean age of 63.5 (±18.1) years were included. Seventy-nine (53.7%) patients failed treatment. Forty-seven (31.9%) patients died within 30 days of onset of MRSA bacteremia. The Charlson index, Pitt bacteremia score and definitive antibiotic regimen were independent factors significantly associated with either treatment failure or mortality. The hVISA phenotype was a potential risk factor predicting treatment failure (adjusted odds ratio 2.420, 95% confidence interval 0.946–6.191, *P* = 0.0652). No bacterial factors were significantly associated with 30-day mortality. In conclusion, the comorbidities, disease severity and antibiotic regimen remained the most relevant factors predicting treatment failure and 30-day mortality in patients with MRSA-RVS bacteremia. hIVSA phenotype was the only bacterial factor potentially associated with unfavorable outcome in this cohort.

## Introduction

MRSA with reduced vancomycin susceptibility phenotype (MRSA-RVS) including heterogeneous vancomycin-intermediate *S*. *aureus* (hVISA) and vancomycin-susceptible *S*. *aureus* (VSSA) with elevated vancomycin MIC (MIC ≥ 1.5 mg/L) have been increasingly reported in the past decade^1–5^. Although the vancomycin MIC of 1.5–2 mg/L remains in the susceptible range, observational studies in the past few years have demonstrated that infections with MRSA-RVS were significantly associated with treatment failure and poor outcomes when treated with vancomycin^5–8^. A meta-analysis showed that high vancomycin MIC was significantly associated with treatment failure irrespective of the source of infection or MIC methodology^9^. Another systemic review further demonstrated that the hVISA phenotype was associated with increased incidence of treatment failure, though the 30-day mortality was similar for hVISA and VSSA infections^10^. These results suggested that the RVS phenotype is an independent factor associated with poor treatment response in patients with MRSA infections.

Intriguingly, it appears that the poor treatment response of patients could not simply be explained by inadequate treatment with vancomycin. Holmes *et al*. demonstrated that RVS phenotype can be significantly associated with increased mortality in patients with methicillin-susceptible *S*. *aureus* bacteremia who were treated with beta-lactams^11^. The underlying mechanism of the association between RVS and poor outcome is not clear and may involve a variety of bacterial genetic or phenotypic features other than drug susceptibility. Understanding of the mechanism will help develop strategies against this medically important pathogen. The accessory gene regulator (*agr)* is a well-studied global regulator of *S*. *aureus*, which controls the expression of a wide array of virulence factors and surface proteins. The *agr* locus was frequently mutated and its function is often compromised in *S*. *aureus* with incremental non-susceptibility to vancomycin^12,13^. The study was aimed to identify factors predicting treatment failure and adverse outcomes in patients with bacteremia caused by MRSA with a vancomycin MIC of 2 mg/L, with focus on a variety of bacterial factors including genotypes, hVISA phenotype and *agr* function.

## Results

### Patient features and outcomes

A total of 147 episodes of bacteremia caused by MRSA with vancomycin MIC of 2 mg/L were identified in 147 patients in this study. The mean ± standard deviation age was 63.5 ± 18.1 years and male gender accounted for 61.2% (90/147) of the cases. Among the147 episodes, 41 (27.9%) episodes were acquired in the community and only 9 (6.12%) episodes occurred in patients without any healthcare-associated risk. Catheter (38.1%) was the most common source of bacteremia. This was followed by pneumonia (23.1%) and skin/soft tissue infections (16.3%). Common comorbidities included renal insufficiency (47.6%), end-stage renal disease requiring dialysis (45.6%), and hypertension (44.9%). Empirical antibiotics with activity against MRSA were administered to 55 (37.4%) cases. Glycopeptides remained to be the most commonly used definitive antimicrobial agent (54.4%, 80/147). Daptomycin was used as the definitive agent in 55(37.4%) of the patients. Sixty-eight patients (46.3%) were treated successfully. The 30-day mortality was 32.0% (47/147) in this cohort. The demographics and clinical features of the 147 patients are summarized in Table 1.

**Table 1:** Univariate analysis of clinical parameters associated with treatment failure and 30-day mortality in 147 episodes of bacteremia due to methicillin-resistant *Staphylococcus aureus* (MRSA) with vancomycin MIC of >1.5 mg/L in a teaching hospital in Taiwan, 2009–2011.

Factor	Total (n = 147)	Treatment response			30-day outcome		
		Failure (n = 79)	Success (n = 68)	P value	Died (n = 47)	Survived (n = 100)	P value
Demographics and medical history							
Female gender (%)	57 (38.8)	30 (38.0)	27 (39.7)	0.8299	18 (38.3)	39 (39.0)	0.9351
Age in years (SD)	63.5 (18.1)	65.7 (16.5)	60.9 (19.5)	0.1078	68.7 (16.2)	61.0 (18.5)	0.0161
Previous *S*. *aureus* infections (%)	68 (46.3)	41 (51.9)	27 (39.7)	0.1393	20 (42.6)	48 (48.0)	0.5368
glycopeptide exposure within 3 mo	61 (41.5)	38 (48.1)	23 (33.8)	0.0798	19 (40.4)	42 (42.0)	0.8566
Community-acquired infections	41 (27.9)	16 (20.3)	25 (36.8)	0.0260	7 (14.9)	34 (34.0)	0.0160
Source of bacteremia (concomitant infections)							
Catheter-associated (%)	56 (38.1)	31 (39.2)	25 (36.8)	0.7579	20 (42.6)	36 (36.0)	0.4454
Bone/joint infections (%)	19 (12.9)	9 (11.4)	10 (14.7)	0.5505	1 (2.13)	18 (18.0)	0.0075
Cardiovascular infections (%)	8 (5.44)	5 (6.33)	3 (4.41)	0.7253^*^	2 (4.26)	6 (6.00)	1.000^*^
Skin and soft tissue infections (%)	24 (16.3)	8 (10.1)	16 (23.5)	0.0284	5 (10.6)	19 (19.0)	0.2008
Pneumonia (%)	34 (23.1)	24 (30.4)	10 (14.7)	0.0246	17 (36.2)	17 (17.0)	0.0101
Intra-abdominal infections (%)	1 (0.68)	0 (0)	1 (1.47)	0.4626^*^	0 (0)	1 (1.00)	1.000^*^
Urinary tract infections (%)	3 (2.04)	0 (0)	3 (4.41)	0.0966^*^	0 (0)	3 (3.00)	0.5514^*^
Unknown (%)	23 (15.7)	13 (16.5)	10 (14.7)	0.7709	10 (21.3)	13 (13.0)	0.1977
Comorbidities							
Charlson index, median (range)	5 (0–14)	6 (0–14)	4 (0–12)	0.0027^#^	6 (0–14)	4 (0–12)	0.0107^#^
DM without end organ damage (%)	21 (14.3)	10 (12.7)	11 (16.2)	0.5433	5 (10.6)	16 (16.0)	0.3863
DM with end organ damage (%)	37 (25.2)	23 (29.1)	14 (20.6)	0.2350	10 (21.3)	27 (27.0)	0.4558
Hypertension (%)	66 (44.9)	32 (40.5)	34 (50.0)	0.2486	17 (36.2)	49 (49.0)	0.1447
Heart failure (%)	26 (17.7)	21 (26.6)	5 (7.35)	0.0023	9 (19.2)	17 (17.0)	0.7501
History of myocardial infarction (%)	6 (4.08)	3 (3.80)	3 (4.41)	1.000^*^	2 (4.26)	4 (4.00)	1.000^*^
Peripheral vascular disease (%)	16 (10.9)	10 (12.7)	6 (8.82)	0.4567	6 (12.8)	10 (10.0)	0.6155
COPD (%)	7 (4.76)	5 (6.33)	2 (2.94)	0.4512^*^	4 (8.51)	3 (3.00)	0.2105^*^
Mild hepatic dysfunction (%)	9 (6.12)	6 (7.59)	3 (4.41)	0.5056^*^	1 (2.13)	8 (8.00)	0.2724^*^
Moderate/severe liver diseases (%)	20 (13.6)	13 (16.5)	7 (10.3)	0.2773	11 (23.4)	9 (9.00)	0.0175
Renal insufficiency (%)	70 (47.6)	40 (50.6)	30 (44.1)	0.4303	21 (44.7)	49 (49.0)	0.6248
On dialysis (%)	67 (45.6)	41 (51.9)	26 (38.2)	0.0972	23 (48.9)	44 (44.0)	0.5752
History of CVA (%)	30 (20.4)	21 (26.6)	9 (13.2)	0.0453	11 (23.4)	19 (19.0)	0.5366
Hemiplegia (%)	25 (17.0)	16 (20.3)	9 (13.2)	0.2588	7 (14.9)	18 (18.0)	0.6401
Dementia (%)	21 (14.3)	12 (15.2)	9 (13.2)	0.7356	7 (14.9)	14 (14.0)	0.8852
Peptic ulcer (%)	23 (15.7)	15 (19.0)	8 (11.8)	0.2294	13 (27.7)	10 (10.0)	0.0060
Immunosuppressive therapy (%)	9 (6.12)	8 (10.1)	1 (1.47)	0.0380^*^	5 (10.6)	4 (4.00)	0.1453
Organ transplant (%)	1 (0.68)	0 (0)	1 (1.47)	0.4626^*^	0 (0)	1 (1.00)	1.000^*^
Connective tissue diseases (%)	2 (1.36)	2 (2.53)	0 (0)	0.4994^*^	1 (2.13)	1 (1.00)	0.5387^*^
Malignant lymphoma/leukemia (%)	4 (2.72)	3 (3.80)	1 (1.47)	0.6241	2 (4.26)	2 (2.00)	0.5930^*^
Tumor without metastasis (%)	15 (10.2)	6 (7.59)	9 (13.2)	0.2600	5 (10.6)	10 (10.0)	1.000^*^
Metastatic solid tumor (%)	20 (13.6)	14 (17.7)	6 (8.82)	0.1167	12 (25.5)	8 (8.00)	0.0038
Surgery requiring hospitalization in previous 30 days (%)	16 (10.9)	8 (10.1)	8 (11.8)	0.7505	4 (8.51)	12 (12.0)	0.5264
Condition at disease onset							
Bacteremia occurring in ICU	35 (23.8)	22 (27.9)	13 (19.1)	0.2153	20 (42.6)	15 (15.0)	0.0003
Pitt bacteremia score, median (range)	2 (0–12)	4 (0–12)	1 (0–7)	<0.0001^#^	5 (0–12)	1 (0–10)	<0.0001^#^
Laboratory data at bacteremia onset							
Hemoglobin, g/dL (SD)	9.60 (1.74)	9.62 (1.83)	9.58 (1.65)	0.8680	9.55 (1.61)	9.63 (1.81)	0.7908
WBC, /μl (SD)	13,489 (8138)	14,145 (8942)	12,756 (7130)	0.3079	15302 (8747)	12665 (7197)	0.1090
Platelet, /μl (SD)	184K (102K)	169K (148K)	201K (114K)	0.0678	158K (101K)	196K (101K)	0.0346
C-reactive protein, mg/L (SD)	118 (82)	125 (83)	111 (114)	0.4702	142 (117)	106 (90)	0.0127
Managements							
Use of permanent catheter	37 (25.2)	23 (29.1)	14 (20.6)	0.2350	12 (25.5)	25 (25.0)	0.9447
Removal of catheter during bacteremia	21 (14.3)	13 (16.5)	8 (11.8)	0.4177	7 (14.9)	14 (14.0)	0.8852
Empirical use of antibiotics with activity against MRSA	55 (37.4)	33 (41.8)	22 (32.4)	0.2393	19 (40.4)	36 (36.0)	0.6051
Definite regimen				0.2645			0.0149
Daptomycin	55 (37.4)	27 (34.2)	28 (41.2)		13 (27.7)	42 (42.0)	
Glycopeptide	80 (54.4)	43 (54.4)	37 (54.4)		26 (55.3)	54 (54.0)	
Others	12 (8.16)	9 (11.4)	3 (4.41)		8 (17.0)	4 (4.00)	

^*^Fisher’s exact test.

^#^Wilcoxon test.

Abbreviations: CVA, cerebral vascular accident; hVISA, heterogeneous vancomycin-intermediate *Staphylococcus aureus*; VSSA, vancomycin-susceptible *S*. *aureus*; COPD, chronic obstructive pulmonary disease; ICU, intensive care unit;.

### Strains characteristics

SCC*mec* type III and *agr* type 1 were the most common genotypes and identified in 105 (71.4%) isolates and 123 (83.7%) isolates, respectively. Community genotypes, including type IV or V SCC*mec*, were identified in 23 (15.6%) isolates. The hVISA phenotype was detected by GRD E-test method in 37.4% of the isolates. The *agr* function was intact in 106 (72.1%) isolates. The detailed characteristics of all isolates are shown in Table 2.

**Table 2:** Univariate analysis of microbiological parameters associated with treatment failure and 30-day mortality in 147 episodes of bacteremia due to MRSA with vancomycin MIC = 2 mg/L in a teaching hospital in Taiwan, 2009–2011.

Factor	Total (n = 147)	Treatment response			30-day outcome		
		Failure (n = 79)	Success (n = 68)	P value	Died	Survived	P value
Resistance to							
Daptomycin (%)	11 (7.48)	8 (10.1)	3 (4.41)	0.1892	4 (8.51)	7 (7.00)	0.7448
Fusidic acid (%)	11 (7.48)	6 (7.59)	5 (7.35)	0.9577	3 (6.38)	8 (8.00)	1.000^*^
Linezolid (%)	0 (0)	0 (0)	0 (0)	…	0 (0)	0 (0)	…
TMP-SXT (%)	108 (73.5)	62 (78.5)	46 (67.7)	0.1380	37 (78.7)	71 (71.0)	0.3226
Teicoplanin (%)	2 (1.36)	0 (0)	2 (2.94)	0.2123	0 (0)	2 (2.00)	1.000^*^
Tigecycline (%)	5 (3.40)	4 (5.06)	1 (1.47)	0.3735	3 (6.38)	2 (2.00)	0.3276
Rifampin (%)	38 (25.9)	23 (29.1)	15 (22.1)	0.3300	16 (34.0)	22 (22.0)	0.1199
hVISA phenotype	55 (37.4)	38 (48.1)	17 (25.0)	0.0039	24 (51.1)	31 (31.0)	0.0191
Delta-hemolysis	106 (72.1)	55 (69.6)	51 (75.0)	0.4683	31 (66.0)	75 (75.0)	0.2542
*Agr* type				0.2533			0.6534
Type 1	123 (83.7)	69 (87.3)	54 (79.4)		41 (87.2)	82 (82.0)	
Type 2	21 (14.3)	10 (12.7)	11 (16.2)		6 (12.8)	15 (15.0)	
Type 4	2 (1.36)	0 (0)	2 (2.94)		0 (0)	2 (2.00)	
untypable	1 (0.68)	0 (0)	1 (2.94)		0 (0)	1 (1.00)	
SCC*mec* type				0.0072			0.2801
II	16 (10.9)	10 (12.7)	6 (8.82)		6 (12.8)	10 (10.0)	
III	105 (71.4)	63 (79.8)	42 (61.8)		37 (78.7)	68 (68.0)	
IV	19 (12.9)	6 (7.59)	13 (19.1)		4 (8.51)	15 (15.0)	
V	4 (2.72)	0 (0)	4 (5.88)		0 (0)	4 (4.00)	
untypable	3 (2.04)	0 (0)	3 (4.41)		0 (0)	3 (3.00)	
PVL genes	1 (0.68)	0 (0)	1 (1.47)	0.4626^*^	0 (0)	1 (1.00)	1.000^*^

^*^Fisher’s exact test.

### Factors associated with treatment failure

The bacterial factors including susceptibilities to various antibiotics, *agr* type and *agr* function were not associated with treatment response (Table 2). Univariate analysis disclosed that the hVISA phenotype was more commonly identified in patients with treatment failure compared to those who were treated successfully (48.1% versus 25.0%, *P* = 0.0039). The distributions of SCC*mec* types also differed in patients with different treatment responses (*P* = 0.0072, Table 2). After adjustment for clinical covariates, the only bacterial factor associated with increased incidence of treatment failure was the hVISA phenotype (adjusted odds ratio [aOR] 2.420, 95% confidence interval [CI] 0.946–6.191) though with marginal significance (*P* = 0.0652). Multivariate analysis revealed that the Charlson index (aOR 1.154, 95% CI 1.009–1.320, *P* = 0.0363) and Pitt bacteremia score (aOR 1.391, 95% CI 1.162–1.666, *P* = 0.0003) were independent clinical factors associated with increased incidence of treatment failure (Table 3).

**Table 3:** Multivariate analysis of factors associated with treatment failure and 30-day mortality in patients with bacteremia due to MRSA with vancomycin MIC of 2 mg/L.

Factor	Treatment failure		30-day mortality	
	aOR (95% CI)	P value	aOR (95% CI)	P value
Age	—	—	1.030 (0.999–1.062)	0.0573
Community-acquired infections	0.762 (0.298–1.946)	0.5694	2.324 (0.646–83.61)	0.1969
Bone/joint infections	–	—	4.834 (0.393–59.438)	0.2184
Skin and soft tissue infections	0.417 (0.136–1.280)	0.1262	—	—
Pneumonia	1.260 (0.435–3.650)	0.6700	1.436 (0.482–4.277)	0.5159
Charlson index	1.154 (1.009–1.320)	0.0363	1.108 (0.951–1.290)	0.1873
Bacteremia occurring in ICU	—	—	2.227 (0.703–7.055)	0.1736
Pitt bacteremia score	1.391 (1.162–1.666)	0.0003	1.298 (1.087–1.549)	0.0040
Platelet	—	—	0.996 (0.991–1.002)	0.2162
C-reactive protein	—	—	1.007 (1.001–1.013)	0.0195
Definite regimen				
daptomycin	—	—	0.128 (0.022–0.746)	0.0286
glycopeptide	—	—	0.238 (0.046–1.246)	0.4597
others	—	—	Referent	—
SCC*mec*				
Type II	Referent	—	—	—
Type III	1.065 (0.278–4.075)	0.9412	—	—
Type IV	1.082 (0.199–5.879)	0.9410	—	—
Type V	—	0.9676	—	—
Non-typable	—	0.9716	—	—
hVISA phenotype	2.420 (0.946–6.191)	0.0652	1.698 (0.623–4.631)	0.3011

### Factors associated with 30-day mortality

Multivariate analysis disclosed that the Pitt bacteremia score and C-reactive protein value at disease onset were independent factors associated with increased incidence of 30-day mortality (aOR 1.298, *P* = 0.0040 and aOR 1.007, *P* = 0.0195, respectively). The use of daptomycin as definitive antimicrobial therapy was independently associated with decreased incidence of 30-day mortality (aOR 0.128, 95% CI 0.022–0.746, *P* = 0.0286). There was no association between bacterial factors and 30-day mortality in this cohort.

## Discussion

The significance of hVISA phenotype on treatment response and patient outcomes had been addressed in previous studies involving bacteremic patients^3,7,13–15^. Data from these studies suggested that the patients with hVISA bacteremia might have higher treatment failure rates compared to those with VSSA bacteremia, The incidence of adverse outcomes were however similar for these infections. In one prospective study Horne *et al*. reported that there is no significant difference in the cure rates of hVISA and VISA infections compared to VSSA infections^8^. The authors concluded that the laboratory identification of the MRSA-RVS might be of limited value and might be reserved for isolates from patients who are failing appropriate anti-MRSA therapy. However, a meta-analysis by van Hal *et al*. found out that the hVISA phenotype was associated with a 2.37-fold increased incidence of treatment failure. There was no association between hVISA and 30-day mortality^10^. Consistent with the observation by van Hal *et al*., results from our study showed that hVISA phenotype was associated with 2.42 folds increased risk of treatment failure. Unfortunately, the difference was of borderline significance which was most likely owing to relative small number of cases in both groups. Together, the difference in patient populations, methods used in the identification of hVISA phenotype (i.e. PAP-AUC, macromethod E-test and GRD E-test), and heterogeneity between patients infected with hVISA and VSSA may be factors responsible for the conflicting results.

It has been shown that MRSA with high vancomycin MIC (i.e. MIC = 2 g/L) was a factor significantly associated with poor outcome in MRSA bacteremic patients^16^. In the current study, in addition to the hVISA phenotype, we failed to identify any bacterial parameters such as antibiotic susceptibilities, SCC*mec* types, *agr* type and *agr* function (δ-hemolysis) that can predict the treatment failure and 30-day mortality when the clinical covariates were adequately controlled. Our data indicated that the clinical condition of the affected patients, particularly the comorbidity and severity at disease onset, remained to be the most critical factor predicting the unfavorable outcomes. This findings supports the current MRSA guideline which stated that the patient’s clinical response should be the primary consideration, irrespective of the MIC^17^.

It was interesting to note that the use of daptomycin as the definitive regimen was associated with decreased incidence of 30-mortality in this MRSA bacteremia cohort. This observation was in agreement with the results in a recent MRSA bacteremia cohort study which demonstrated a decrease 30-day mortality and persistent bacteremia in patients on early use of daptomycin^18^. However, this observation should be interpreted with caution since our study was not designed to address the efficacy of different therapeutic regimen on MRSA bacteremia. The significance of the role of daptomycin in decreased 30-day mortality may be due to other factors that were not considered in this study. For instance, daptomycin was usually used as an alternative agent to glycopeptide in MRSA bacteremic patients. It was likely that those survived 30 days after disease onset might have greater chance of receiving daptomycin treatment. Nevertheless, given the high incidence of case fatality and the potential benefit of this agent in reducing mortality, the early use of antimicrobial agents alternative to glycopeptides should be seriously considered in those with multiple comorbidity and severe infections at disease onset (i.e. high Pitt bacteremia score and CRP).

There were limitations of the study. Firstly, we found out that there was a huge heterogeneity of clinical parameters between the two groups of patients. Heterogeneity of patient characteristics, thought having taken into consideration and adjusted by multivariate logistic regression method, might still exist and potentially skew our findings. A prospective randomized control study with sufficient number of participants will be the ultimate way to clarify the issue addressed in this study. Secondly, the sample size of the study was relative small which might not be able to precisely estimate the impact of certain clinical or bacterial parameters.

In conclusion, bacteremia due to MRSA with high vancomycin MIC is associated with extremely high incidences of treatment failure and adverse outcomes. Our data indicated that the underlying conditions, severity of infection at disease onset and antibiotic regimen remained the most critical factors predicting the patients’ outcomes. Our data also suggested that the hVISA phenotype was the only potential factor predicting treatment failure in this population. Based on these findings, we agree that detection of the hVISA phenotype might of clinical relevance and routine laboratory detection might be considered^8,19^.

## Methods

### Ethic statements

This study was approved by the institutional review boards (IRB) of Chang Gung Memorial Hospital at Linkou (GMH-Linkou). All experimental protocols were performed in accordance with the guidelines and regulations of the IRB of CGMH. A waiver of consent was granted given the retrospective nature of the project and anonymous analysis of the clinical information.

### Study design

The study was conducted in a university-affiliated teaching hospital (CGMH-Linkou) in northern Taiwan from August 2009 to July 2012. The hospital is a 3,715-bed medical center and provided both primary and tertiary healthcare. It consists of 13 disease-oriented departments, including 26 intensive care units (ICUs) and 73 ordinary wards. A central microbiology laboratory was responsible for processing all clinical specimens. From August 01 2009, the microbiology laboratory began to report the vancomycin MICs values for invasive *S*. *aureus* isolates (from sterile sites, i.e. blood). The MICs were determined by the E-test method (BioMerieux, France). The *S*. *aureus* isolates were stored at −80 °C after their isolation and were accompanied by an electronic record containing the information of the isolates and the chart number of patients. We reviewed the clinical information of patients with MRSA bacteremia with a vancomycin MIC = 2 mg/L.

### Demographics and anthropometric variables of subjects

Only the initial episodes of bacteremia during the study period was included for analysis to preserve the independence of observations. Positive blood cultures obtained from patients without consistent or persistent features of systemic infections were considered to have been either contaminated or transient bacteremia. Data for such patients were excluded from analysis.

A standardized data collection form was used to collect the medical information needed in this study (Table 1). We identified the possible sources of bacteremia (concomitant infections), comorbid illnesses, concurrent blood isolates of other bacterial species, clinical condition within 48 hours or on the day of onset of bacteremia, laboratory and image findings, ICU stay, empiric and definite antimicrobial regimens and a variety of unfavorable outcomes. Renal insufficiency was defined as a serum creatinine level ≥ 1.4 mg/dL without the requirement of hemodialysis. The sources of bacteremia were identified by reviewing the medical records, radiologic studies, surgical findings and microbiological records of the patients. The Charlson weighted index of comorbidity (WIC) and Pitt bacteremia score were calculated and used as parameters to control for comorbidity and severity of illness at the onset of bacteremia during the analysis of risks associated with treatment failure and 30-day mortality.

Appropriate definitive antimicrobial treatment was defined as antibiotics active against the offending pathogens started within 48 hours of onset of bacteremia and used for at least 3 days. Adjunctive therapy was defined as concurrent surgical intervention or palliative drainage. Treatment failure was defined as inadequate response to therapy, such as the development of resistance to glycopeptide; worsening, recurrent or new onset of signs and symptoms requiring a change of antibiotic regimen; or a positive blood culture for MRSA at the end of therapy. Mortality occurring within 7 days of *S*. *aureus* bacteremia was defined as bacteremia-attributed mortality if there was no other identified cause for death. The 30-day mortality was defined as any cause of death within 30 days of MRSA bacteremia onset.

### Determination of hVISA phenotype

Etest GRD (bioMérieux, Marcy-l’Etoile, France) was used to determine the hVISA phenotype and performed according to manufacturer’s instructions^20^. Briefly, a bacterial suspension at a 0.5 McFarland standard was inoculated onto a MHA plate containing 5% sheep blood (BBL; Becton Dickinson, Cockeysville, MD) and incubated at 35 °C. The zone of inhibition was read at 24 and 48 h after incubation. An isolate was considered hVISA if the Etest GRD strip result was ≥8 mg/L for vancomycin or teicoplanin.

### δ-Hemolysin production

Delta-hemolysin production is indicative of intact *agr* function. This was measured according to the procedure described elsewhere^19^. Briefly, the *S*. *aureus* isolates were streaked perpendicularly to RN4220 on Columbia agar plates (Oxoid, Cambridge, UK) with 5% sheep blood and incubated at 37 °C in 5% CO_2_ overnight. Synergistic hemolysis within the *β*-hemolysin zone produced by RN4220 was evaluated. The presence of synergistic hemolysis indicates the production of δ-hemolysin. ATCC 25923 and Mu50 were used as positive and negative controls, respectively, for the production of δ-hemolysin.

### Genotyping of MRSA strains

Staphylococcal cassette chromosome *mec* element (SCC*mec*) typing of MRSA isolates was performed using a multiplex PCR strategy described in a previous study^14^. The control strains for SCC*mec* types I, II, III, IVa, and V_T_ were *S*. *aureus* NCTC10442, N315, 85/2082, JCSC4744 and TSGH-17, respectively. SCC*mec* typing was determined by using a particular primer described elsewhere^21^. The presence of Panton-Valentine leukocidin (PVL) genes was determined in all *S*. *aureus* isolates by a PCR technique described by Lina *et al*.^22^. The *agr* type was determined as previously described^23^. The *agr* sequences were amplified with the following primers, Pan (5′-ATGCACATGGTGCACATGC-3′), *agr*1 (5′-GTCACAAGTACTATAAGCTGCGAT-3′), *agr*2 (5′-TATTAC TAATTGAAA AGTGGCCATAGC-3′), *agr*3 (5′-GTAATGTAATAGCTT GTATAATAATA CCCAG-3′) and *agr*4 (5′-CGATAATGCCGT AATACCCG-3′). These primers allowed amplification of a 441-bp DNA fragment from *agr* group I, 575-bp fragment from *agr* group II, 323-bp fragment from *agr* group III, and 659-bp DNA fragment from *agr* group IV strains.

### Statistical analysis

Comparison of categorical variables between study groups was performed with a chi-square test or with the Fisher exact test where appropriate, whereas differences among the numerical variables were analyzed by two-sample *t*-test. Multiple logistic regression analysis was applied to explore factors associated with treatment failure and 30-day mortality. Statistic significance was defined as a *P* value of <0.05 in the tests. The statistics were performed using an SAS 9.3 for windows (SAS Institute, Inc., Cary, NC).
